# Supplementary material for: A fault-intrusion-tolerant system and deadline-aware algorithm for scheduling scientific workflow in the cloud
Source: PeerJ Comput Sci. 2021 Nov 2;7:e747. doi: 10.7717/peerj-cs.747 (PMC8576554; doi:10.7717/peerj-cs.747)
Supplement: Supplemental Information 6 [file peerj-cs-07-747-s006.zip › Myproject1.1/docs/org/cloudbus/cloudsim/power/models/package-summary.html]

org.cloudbus.cloudsim.power.models


JavaScript is disabled on your browser.


- Overview
- Package
- Class
- Use
- Tree
- Deprecated
- Index
- Help

- Prev Package
- Next Package

- Frames
- No Frames

- All Classes

# Package org.cloudbus.cloudsim.power.models

- Interface Summary

  | Interface | Description |
  | --- | --- |
  |  |  |
  | --- | --- |
  | PowerModel | The PowerModel interface needs to be implemented in order to provide a model of power consumption depending on utilization for system components. |
- Class Summary

  | Class | Description |
  | --- | --- |
  |  |  |
  | --- | --- |
  | PowerModelCubic | The Class PowerModelCubic. |
  | PowerModelLinear | The Class PowerModelLinear. |
  | PowerModelSpecPower | The abstract class of power models created based on data from SPECpower benchmark: http://www.spec.org/power\_ssj2008/ If you are using any algorithms, policies or workload included in the power package, please cite the following paper: Anton Beloglazov, and Rajkumar Buyya, "Optimal Online Deterministic Algorithms and Adaptive Heuristics for Energy and Performance Efficient Dynamic Consolidation of Virtual Machines in Cloud Data Centers", Concurrency and Computation: Practice and Experience (CCPE), Volume 24, Issue 13, Pages: 1397-1420, John Wiley & Sons, Ltd, New York, USA, 2012 |
  | PowerModelSpecPowerHpProLiantMl110G3PentiumD930 | The power model of an HP ProLiant ML110 G3 (1 x [Pentium D930 3000 MHz, 2 cores], 4GB). |
  | PowerModelSpecPowerHpProLiantMl110G4Xeon3040 | The power model of an HP ProLiant ML110 G4 (1 x [Xeon 3040 1860 MHz, 2 cores], 4GB). |
  | PowerModelSpecPowerHpProLiantMl110G5Xeon3075 | The power model of an HP ProLiant ML110 G5 (1 x [Xeon 3075 2660 MHz, 2 cores], 4GB). |
  | PowerModelSpecPowerIbmX3250XeonX3470 | The power model of an IBM server x3250 (1 x [Xeon X3470 2933 MHz, 4 cores], 8GB). |
  | PowerModelSpecPowerIbmX3250XeonX3480 | The power model of an IBM server x3250 (1 x [Xeon X3480 3067 MHz, 4 cores], 8GB). |
  | PowerModelSpecPowerIbmX3550XeonX5670 | The power model of an IBM server x3550 (2 x [Xeon X5670 2933 MHz, 6 cores], 12GB). |
  | PowerModelSpecPowerIbmX3550XeonX5675 | The power model of an IBM server x3550 (2 x [Xeon X5675 3067 MHz, 6 cores], 16GB). |
  | PowerModelSqrt | The Class PowerModelSqrt. |
  | PowerModelSquare | The Class PowerModelSquare. |

- Overview
- Package
- Class
- Use
- Tree
- Deprecated
- Index
- Help

- Prev Package
- Next Package

- Frames
- No Frames

- All Classes
